# Aberrant myocardial calcium cycling: pathogenic mechanisms and targeted therapeutics for heart failure

**DOI:** 10.3389/fcvm.2026.1938059

**Published:** 2026-09-03

**Authors:** Chunxiao Zhao, Jiahao Liu, Juanjuan Xin, Qun Liu, Ying Zhang, Xiaochun Yu, Junhong Gao

**Affiliations:** 1 The Affiliated Hospital of Shandong University of Traditional Chinese Medicine, Jinan, Shandong, China; 2 Shandong University of Traditional Chinese Medicine, Jinan, Shandong, China; 3 Institute of Acupuncture and Moxibustion, China Academy of Chinese Medical Sciences, Beijing, China; 4 Jinan Municipal Government Hospital, Jinan, Shandong, China

**Keywords:** autonomic neural regulation, calcium cycling, calcium cycling-related proteins, excitation-contraction coupling, heart failure

## Abstract

Cardiovascular diseases rank as the global primary cause of mortality. Heart failure (HF) represents a progressive cardiac syndrome triggered by structural or functional myocardial damage that compromises ventricular systolic output. Intracellular calcium homeostasis underpins cardiomyocyte excitation-contraction coupling. Calcium overload and dysregulated calcium handling act as central pathogenic forces driving HF progression. Sympathetic β_1_-adrenergic receptor (β_1_-AR) and parasympathetic M_2_ muscarinic acetylcholine receptor (M_2_-AChR) coordinate dual regulatory signaling to control cardiac calcium turnover, alongside key modulators including L-type calcium channel (LTCC), ryanodine receptor 2 (RyR2), SERCA2a, cardiac troponin C (cTnC), Na^+^/Ca^2+^ exchanger 1 (NCX1) and mitochondrial permeability transition pore (MPTP). This review outlines physiological calcium cycling and systematically characterizes HF-related functional defects of calcium regulatory machinery from three layers: calcium release, sarcoplasmic reticulum calcium reuptake and auxiliary calcium mediators. We further discuss the underlying mechanisms of calcium-targeted pharmaceuticals and gene therapeutic approaches, while addressing translational bottlenecks limiting single-target interventions. Restoration of balanced cytosolic calcium cycling holds therapeutic potential to reverse pathological myocardial remodeling in HF.

## 1 Introduction

Cardiovascular diseases account for the greatest global mortality burden. Heart failure (HF) is a progressive multifactorial clinical syndrome. It arises from structural or functional myocardial damage that impairs ventricular contractile performance and is associated with poor long-term outcomes; only 35% of newly-diagnosed patients survive beyond five years (1) Population-based epidemiological investigations demonstrate HF affects approximately 2% of the adult population, with prevalence rising to 5%–9% in adults aged above 65 (2). Nationwide cohort studies from China report a national HF prevalence of 1.1%, corresponding to roughly 12.1 million patients. Among these patients, 40.5% experience at least three hospitalizations each year (3). Notably, levosimendan, a calcium sensitizer, obtained EU marketing authorization in the early 2000s. Since then, no novel therapies directly targeting myocardial calcium handling have been approved for HF for more than two decades (4). This unmet need defines an urgent translational gap, which the present review seeks to address. Therapeutic choices for advanced HF are still limited. Coupled with heavy economic burdens, HF represents a major public-health priority.

Sustained cytosolic Ca^2+^ homeostasis and intact calcium cycling underpin cardiomyocyte excitation-contraction (E-C) coupling, and well-coordinated systolic and diastolic cardiac function. Accumulated evidence identifies calcium overload and deranged calcium handling as central pathogenic factors driving HF progression (5). Defective E-C coupling is a hallmark of the failing myocardium. Calcium release and reuptake are two indispensable components of this coupling process. Remodeling of calcium-regulatory proteins, both structural and functional, triggers impaired myocardial contractility at the molecular level.

## 2 Physiological patterns of cardiac calcium cycling

Ca^2+^ serves as an essential mediator governing cardiomyocyte electrophysiological behavior and contractile function. Dynamic shifts in intracellular Ca^2+^ concentration originate from membrane depolarization induced by action potentials, laying the molecular groundwork for myocardial contraction (6). Cardiac E-C coupling describes the conversion of electrical excitation into mechanical contraction within cardiomyocytes, where Ca^2+^ functions as a vital secondary messenger. Normal cardiac function relies entirely on intact calcium cycling to sustain E-C coupling, a process regulated reciprocally by sympathetic and parasympathetic neural signaling (7).

The sarcoplasmic reticulum (SR) functions as the primary intracellular Ca^2+^ store, controlling Ca^2+^ efflux during systole and calcium recycling throughout diastole. Under physiological sympathetic stimulation, nerve terminals release norepinephrine (NE), which binds membrane-localized β_1_-adrenergic receptor (β_1_-AR) and initiates canonical intracellular signaling cascades (8). This signaling cascade activates stimulatory G protein (Gs), which catalyzes the conversion of guanosine diphosphate (GDP) to guanosine triphosphate (GTP). GTP-bound activated Gs further stimulates adenylate cyclase (AC) to convert adenosine triphosphate (ATP) into cyclic adenosine monophosphate (cAMP), subsequently triggering protein kinase A (PKA) activation. PKA catalyzes the phosphorylation of L-type calcium channels (LTCCs), enabling minor extracellular Ca^2+^ influx. This subtle Ca^2+^ entry activates sarcoplasmic RyR2 channels, triggering massive Ca^2+^ release from SR terminal cisternae into the cytosol. Elevated cytosolic Ca^2+^ binds cardiac troponin C (cTnC) and induces conformational rearrangement, displacing tropomyosin and unmasking active binding sites on actin filaments. This event drives cross-bridge cycling between thick and thin myofilaments and initiates cardiomyocyte contraction (9). Accordingly, cTnC is recognized as the molecular switch that modulates myocardial contractile activity.

During diastole, Ca^2+^ dissociates from cTnC and is reimported into the SR via membrane-embedded SERCA2a to replenish calcium stores for subsequent systolic release (10). Declining cytosolic Ca^2+^ levels facilitate myocardial relaxation. SERCA2a activity is persistently suppressed by membrane-bound phospholamban (PLB). This cyclic cytosolic Ca^2+^ release and reuptake constitutes the definition of cardiac calcium cycling, which acts as the central framework maintaining intact E-C coupling and coordinated cardiac contraction-relaxation cycles. Beyond SR-dependent calcium turnover, stable cytosolic Ca^2+^ homeostasis is jointly maintained by Na^+^/Ca^2+^ exchanger 1 (NCX1) (11) and the mitochondrial permeability transition pore (MPTP) (12). Without Ca^2+^ acting as a signaling intermediate, cardiomyocyte electrical excitation fails to couple with mechanical shortening, generating action potentials unaccompanied by myocardial contraction.

Upon parasympathetic nerve activation, nerve terminals secrete acetylcholine (ACh) to bind cardiac M_2_ acetylcholine receptor (M_2_-AChR). Signaling cascades downstream of inhibitory G protein (Gi) exert inhibitory effects on cardiac function and counterbalance excessive sympathetic stimulation (13). Under resting physiological conditions, sympathetic signaling dominates cardiac regulatory networks, while parasympathetic input restrains overactive sympathetic tone to elicit negative inotropic and chronotropic responses.

HF drives widespread remodeling of cardiomyocyte calcium regulatory networks, a process tightly linked to systolic dysfunction and arrhythmogenesis (14). Defective E-C coupling in failing myocardium correlates with altered expression levels and functional activity of calcium regulatory proteins (15). Multiple pathological stimuli including myocardial ischemia and hypertension trigger cardiac remodeling and impaired relaxation, two hallmark pathological phenotypes of HF (16). Excessively activated sympathetic signaling in HF elevates catecholamine concentrations and disrupts downstream β_1_-AR signaling cascades, triggering cytosolic calcium overload, myocardial injury and arrhythmia. One representative calcium cycling defect in failing hearts is aberrant RyR2 activity. Although impaired interaction with calstabin (FKBP12.6) has been proposed to contribute to RyR2 dysfunction, this mechanistic interpretation remains debated within the field (17). Pathological RyR2 dysfunction gives rise to diffuse spontaneous diastolic SR Ca^2+^ leak originating from stochastic opening of individual RyR2 channels. Importantly, diastolic SR Ca^2+^ leak should not be equated with Ca^2+^ sparks. Ca^2+^ sparks represent discrete, spatially-localized Ca^2+^ release events triggered by coordinated opening of RyR2 channel clusters (18). In heart failure, pathological RyR2 hyperactivity may increase both diffuse diastolic SR Ca^2+^ leak and the frequency of Ca^2+^ sparks. Chronic β_1_-adrenergic over-stimulation under HF conditions promotes RyR2 hyperphosphorylation at Ser2808 (PKA-dependent) and Ser2814 (CaMKII-dependent); phosphorylation at Ser2814 is particularly critical for β-adrenergic-stress-provoked SR Ca^2+^ leak (19–21). It should be noted that the regulation of these two phosphorylation sites is complex, since genetic ablation of both residues does not uniformly abolish pro-arrhythmic spontaneous Ca^2+^ release (22). The diastolic SR Ca^2+^ leak can activate NCX1, generating inward electrogenic currents that produce delayed afterdepolarisations (DADs) and constitute key arrhythmogenic triggers (23).

Such calcium leakage depletes SR calcium reserves and diminishes myocardial contraction amplitude (24). Meanwhile, calcium oscillations arising from leakage serve as major triggers for arrhythmias linked to delayed afterdepolarization (DAD) (25). Another prevalent defect in calcium handling involves reduced SERCA2a expression and suppressed activity, accompanied by diminished PLB phosphorylation. This change amplifies PLB's inhibitory influence over SERCA2a, lowering diastolic Ca^2+^ reuptake and impairing myocardial relaxation (26, 27). Blunted responsiveness of β_1_-AR to catecholamines also contributes to weakened systolic performance (28). Accumulated experimental evidence demonstrates that cardiomyocytes isolated from failing hearts exhibit reduced Ca^2+^ sensitivity, reflected by weakened binding affinity and unstable interaction between cTnC and cytosolic Ca^2+^ (29). Furthermore, functional impairment of NCX1 (30) and MPTP (31) disrupts cytosolic Ca^2+^ balance and exacerbates cardiomyocyte damage. Depleted SR calcium storage arises from three concurrent alterations: enhanced NCX1 activity, suppressed SERCA2a function and sustained RyR2-mediated calcium leakage. Taken together, all these disruptions to calcium signaling give rise to combined systolic and diastolic dysfunction in HF. Meanwhile, parasympathetic signaling becomes impaired in HF and loses its inhibitory constraint on over-activated sympathetic activity, inducing a spectrum of electrical and mechanical cardiac abnormalities and accelerating disease progression (32).

**Figure 1:**
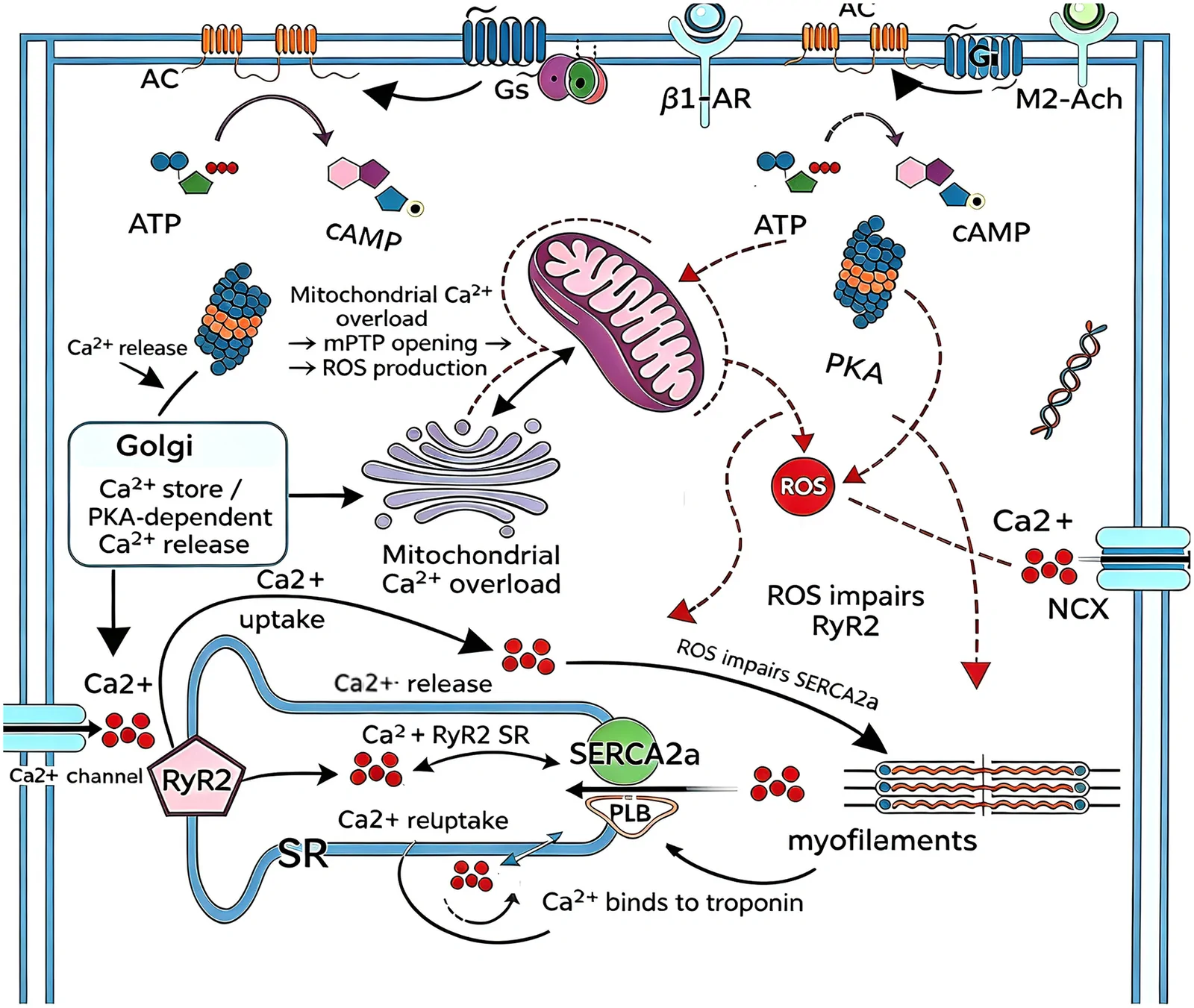
Schematic illustration of β_1_-AR and M_2_-AChR signaling cascades linked to cardiomyocyte E-C coupling. This schematic illustrates dual autonomic neural regulation of cytosolic calcium cycling in ventricular cardiomyocytes. Sympathetic stimulation triggers the Gs–AC–cAMP–PKA cascade, which enhances LTCC-dependent Ca^2+^ entry and RyR2-driven sarcoplasmic reticulum (SR) calcium release to facilitate myocardial contraction. Parasympathetic signaling activates inhibitory Gi proteins to restrain cAMP production and counteract the positive inotropic actions of sympathetic activation. The SERCA2a–PLB complex drives diastolic Ca^2+^ reuptake into the SR, whereas NCX1 primarily extrudes cytosolic Ca^2+^ under physiological conditions. Mitochondrial compartments and myofilament structures are annotated to present the complete intracellular calcium signaling network, including mitochondrial permeability transition pore (MPTP)-associated calcium handling.

In summary, calcium cycling participates in every stage of cardiomyocyte E-C coupling. Sympathetic β_1_-AR and parasympathetic M_2_-AChR signaling pathways coordinate to modulate calcium homeostasis and sustain cardiac function (Figure 1). The key molecular players involved in this process, along with their regulatory mechanisms and pathological alterations in heart failure, are summarized in Table 1.

**Table 1:** Calcium handling proteins and their modulators in HF.

Protein/Complex	Alterations in HF	Functional impact	Representative modulators
LTCC (Cav1.2, *α*_1_C subunit)	Reduced t-tubule density; Increased open probability	Impaired CICR; microdomain uncoupling	Roscovitine (Selective late Ca^2+^ current inhibitor, no negative inotropy); Verapamil (Non-selective L-type blocker)
RyR2-Calstabin2; IP3R	PKA/CaMKII-dependent hyperphosphorylation; Calstabin2 dissociation; Interdomain destabilization; Elevated IP3R expression with enhanced IP3-dependent SR Ca^2+^ release	Diastolic SR Ca^2+^ leak; Arrhythmogenic DADs; Exacerbated by sympathetic overactivation; IP3R mediates slow sustained SR Ca^2+^ efflux under Gq-coupled signaling, augmenting diastolic Ca^2+^ overload and providing Ca^2+^ substrate for mitochondrial calcium uptake	TV519 (Stabilizer; Phase II halted); Dantrolene (RyR-stabilizer; reduces diastolic SR Ca^2+^ leak; pre-clinical); Phenytoin (decreases RyR2 open probability; antiarrhythmic, pre-clinical); Flecainide (RyR2/Na^+^-channel blocker; clinically applied in CPVT); β-blockers (Suppress PKA); FKBP12.6 gene therapy; 2-APB, Xestospongin C (IP3R antagonists, pre-clinical)
SERCA2a-PLB	Reduced SERCA2a activity; PLB hypophosphorylation	Slowed diastolic Ca^2+^ reuptake; Prolonged Ca^2+^ transients; Impaired relaxation	Istaroxime (PLB inhibitor); Ginsenoside Rg3 (SUMOylation; low toxicity); AAV1.SERCA2a (Trial failed)
cTnC	Reduced Ca^2+^ binding affinity; Altered EF-hand domain conformation	Blunted contraction; Ca^2+^-force discordance	Levosimendan (Ca^2+^ sensitizer); Omecamtiv mecarbil (Myosin activator, indirect cTnC modulation)
NCX1	Upregulated transcription/translation; Enhanced reverse-mode activity; Increased Na^+^-dependent Ca^2+^ influx	Cytosolic Ca^2+^ overload; Depleted SR Ca^2+^ stores; Pro-arrhythmic	SEA0400 (Selective NCX inhibitor; Suppresses reverse-mode Ca^2+^ influx; Reduces ventricular fibrosis in preclinical HF models)
MPTP; mtCU complex (MCU, EMRE, MICU1, MICU2); NCLX	Increased opening susceptibility to Ca^2+^ overload/oxidative stress; Cyclophilin D activation; mtCU remodeling with altered MICU1/MICU2 stoichiometry; suppressed NCLX-mediated Ca^2+^ efflux due to high intracellular Na^+^	Mitochondrial depolarization; ATP depletion; Initiation of apoptosis/necrosis; Exacerbated by cytosolic Ca^2+^ excess; Dysbalanced mtCU-NCLX activity causes mitochondrial matrix Ca^2+^ overload, increases ROS production and lowers MPTP activation threshold	Cyclosporine A (Inhibits cyclophilin D; Reduces infarct size in acute MI; Potential for chronic HF remodeling); Antioxidants (Indirect MPTP inhibition via ROS scavenging); Ru360 (mtCU inhibitor); CGP-37157 (NCLX partial inhibitor, pre-clinical)

cTnC, cardiac troponin C; CICR, calcium-induced calcium release; DADs, delayed afterdepolarizations; CaMKII, calcium/calmodulin-dependent protein kinase II; LTCC Cav1.2, L-type calcium channel; MI, myocardial infarction; MPTP, mitochondrial permeability transition pore; NCX1, Na^+^/Ca^2+^ exchanger 1; PKA, protein kinase A; PLB, phospholamban; ROS, reactive oxygen species; SERCA2a, sarco/endoplasmic reticulum Ca^2+^-ATPase 2a; SR, sarcoplasmic reticulum; IP3R, inositol 1,4,5-trisphosphate receptor; mtCU, mitochondrial calcium uniporter complex; NCLX, mitochondrial Na^+^-Ca^2+^-Li^+^ exchanger; MCU, pore-forming subunit of mtCU; EMRE, essential MCU regulator; MICU1/2, mitochondrial calcium uptake 1/2.

## 3 Sympathetic and parasympathetic target receptors: β1-AR and M2-AChR

### 3.1 β1-Adrenergic receptor (β1-AR)

Cardiac β-adrenergic receptors (β-ARs) are divided into three distinct subtypes: β_1_-AR, β_2_-AR and β₃-AR. Each subtype couples with distinct G-protein families to activate unique intracellular signaling cascades in response to sympathetic stimulation (8). β-AR signaling serves as an essential modulator of cardiac output. Under sustained sympathetic activation, β-ARs translocate from the plasma membrane to intracellular endosomal compartments, reducing the number of membrane-resident receptors available for signal transduction. This adaptive process is widely recognized as β-AR downregulation (33). As the predominant cardiac subtype, β_1_-AR accounts for 70%–80% of total myocardial β-AR expression and primarily modulates heart rate and contractile function. Dysfunction of β_1_-AR signaling is closely implicated in multiple cardiovascular diseases. Accumulating evidence indicates that persistent agonist exposure, such as isoproterenol (ISO) stimulation, induces β_1_-AR internalization and perinuclear translocation (34). Following agonist withdrawal, internalized β_1_-ARs can recycle back to the cell membrane independently of protein degradation, thereby modulating adenylate cyclase (AC) activity (34, 35).

Heart failure (HF) is characterized by persistent sympathetic overactivation, which induces chronic β_1_-AR stimulation via sustained catecholamine release (36). Catecholamine binding to β_1_-AR triggers the downstream AC–cAMP–PKA signaling cascade. In healthy hearts, acute sympathetic activation enhances cardiac contractility and heart rate to adapt to physiological demands. In contrast, prolonged β_1_-AR stimulation in failing hearts drives pathological cardiac remodeling and impairs β-adrenergic signal transduction, ultimately blunting myocardial contractile responses (37). Chronic β_1_-AR overstimulation induces cardiomyocyte apoptosis and progressive left ventricular remodeling. In adult zebrafish HF models, prolonged ISO exposure for 14 days significantly upregulates both β_1_-AR and β_2_-AR expression, leading to severe contractile impairment, β_1_-AR desensitization, increased cardiomyocyte death, elevated BNP secretion, and disrupted calcium homeostasis (38). Consistently, cardiomyocyte-specific β_1_-AR overexpression in transgenic mice results in myocardial fibrosis, compromised cardiac function, and increased age-associated mortality (39). Multiple studies have further confirmed that sustained β_1_-AR hyperstimulation, either by ISO administration or genetic overexpression, exacerbates myocardial necrosis and interstitial fibrosis by promoting extracellular calcium influx (40, 41). With HF progression, persistent sympathetic hyperactivity aggravates pathological β_1_-AR desensitization and weakens myocardial responsiveness to catecholamines (42).

Notably, substantial mechanistic findings regarding β-AR remodeling have been validated in human atrial and ventricular myocardium from both non-failing and failing hearts. Human tissue studies have verified profound β_1_-AR downregulation, receptor-G protein uncoupling, and functional desensitization in failing human myocardium. Unlike β_1_-AR, β_2_-AR undergoes complex functional remodeling rather than simple downregulation under HF conditions (43, 44). Further studies indicate that failing human cardiomyocytes exhibit rearranged subcellular cAMP microdomains and altered local β-AR-RyR2 signaling, which facilitates the formation of calcium-dependent arrhythmogenic substrates (45, 46). Even desensitized β-adrenergic signaling retains the capacity to induce cytosolic calcium overload through abnormal activation of downstream LTCC and RyR2 activity (47).

In addition to β_1_-AR-mediated pathological remodeling, β_2_-AR signaling exerts critical proarrhythmic effects in the remodeled failing human heart. Under heart-failure conditions, β_2_-AR stimulation can elicit more prominent arrhythmic responses compared with β_1_-AR activation in human cardiac tissue preparations (48). Mechanistically, β_2_-AR signaling enhances Ca^2+^ transient amplitude and sarcoplasmic reticulum Ca^2+^ load, and increases the transmural dispersion between action potential duration and Ca^2+^ transient duration. Such electrophysiological remodeling promotes spontaneous SR Ca^2+^ release, delayed afterdepolarizations, and subsequent triggered arrhythmias (48, 49).

Given the above sympathetic-driven pathological alterations in HF, β-blockers serve as the cornerstone therapeutic strategy for systolic heart failure. These agents suppress excessive sympathetic tone and alleviate catecholamine-induced myocardial injury. Preclinical studies have demonstrated that the selective β_1_-AR antagonist metoprolol effectively inhibits cardiomyocyte apoptosis, reduces the expression of pro-inflammatory factors including TNF-α and IL-1β, and attenuates adverse left ventricular remodeling (50). Selective β_1_-blockers exert consistent protective effects in both juvenile and adult ISO-induced murine HF models, while non-selective β-blockers ameliorate pathological cardiac hypertrophy and collagen deposition (51). Clinical trial outcomes corroborate the clinical value of β-blockers: selective bisoprolol substantially reduces mortality and cardiovascular event risk among patients with HF with reduced ejection fraction, and early intravenous administration yields sustained long-term prognostic benefits for individuals with severe left ventricular systolic dysfunction (52, 53). Collectively, β-blockers mitigate catecholamine-associated myocardial damage, suppress β_1_-AR-mediated apoptotic signaling, slow progressive ventricular remodeling, and improve long-term prognosis, making them indispensable first-line agents for routine HF clinical treatment.

### 3.2 Muscarinic acetylcholine receptor subtype 2 (M2-AChR)

Muscarinic cholinergic receptors (M receptors) consist of five distinct subtypes (M_1_–M_5_) that mediate extensive central and peripheral physiological activities, serving as high-priority intervention targets for multiple cardiovascular disorders (54). Transcriptomic data indicate abundant M_2_ mRNA expression within rat atrial and ventricular myocardium, accounting for over 90% of total cardiac muscarinic receptor transcripts, with uneven tissue distribution: atrial M_2_ expression reaches twice that measured in ventricular tissue (55). Although low levels of M_1_ receptor transcripts are detectable in ventricular tissue (56), M_2_-AChR represents the predominant parasympathetic receptor in cardiomyocytes. It couples to inhibitory Gi proteins to trigger negative chronotropic and negative inotropic responses. Atrial M_2_ receptor gene directly suppresses contractile amplitude, whereas ventricular M_2_ signaling generates indirect negative inotropic effects only when myocardial contractility is elevated by β-adrenergic agonists (57).

Emerging evidence demonstrates elevated M_2_ receptor density in failing myocardium. This upregulation enhances cellular sensitivity to cholinergic agonists yet blunts contractile responses triggered by β-adrenergic stimulation (58). Upregulated cardiac M receptors exert bidirectional effects under HF conditions: cholinergic signaling counteracts β-mediated enhancement of myocardial contraction, while attenuated adrenergic depolarization confers cardioprotection and reduces ventricular arrhythmia risk. Several groups report only mild functional modifications of M_2_ receptors under pathological stress or pharmacological intervention (59). Sustained reduction in parasympathetic tone impairs M_2_ receptor function and aggravates autonomic nervous system imbalance. M_2_-AChR signaling mediates vagally induced bradycardia, a phenotype completely abolished in M_2_ knockout murine models (60). Following acute pharmacological blockade of cardiac muscarinic receptors with atropine, β_1_-AR-dependent positive chronotropic effects are markedly potentiated, confirming that endogenous parasympathetic tone restrains excessive β-adrenergic signaling. M_2_-AChR signaling confers intrinsic cardioprotective properties: compared with wild-type littermates, M_2_ knockout mice display heightened stress susceptibility and aggravated ventricular dysfunction under ISO stimulation (61). Zebrafish-based studies further validate that hypoxic bradycardia relies on intact cardiac M_2_-AChR signaling, and M_2_ gene knockdown eliminates this physiological response in larval fish (62).

Notably, M_2_ receptor activation elicits unique positive inotropic effects in ventricular myocardium isolated from failing hearts. Functional experiments confirm that carbachol (CCH)-stimulated M_2_ signaling elevates myosin light chain phosphorylation, augments myofilament Ca^2+^ sensitivity, and generates net positive contractile responses in ventricular tissues from HF rats (63). Subsequent mechanistic research reveals CCH facilitates reverse-mode transport of NCX1 to amplify cytosolic calcium transients, with M_2_-AChR participating in this regulatory cascade (64). These findings position M_2_-AChR as a key node linking parasympathetic tone to calcium handling, complementing the well-characterized sympathetic-β_1_-AR axis in HF pathophysiology and highlighting its potential as a therapeutic target to restore autonomic-calcium balance. Beyond intrinsic cardiac regulation, cerebellar M_2_ receptors also participate in systemic cardiovascular homeostasis. The selective M_2_ agonist Arecaidine But-2-ynyl Ester Tosylate (ABET) lowers mean arterial pressure and heart rate in a dose-dependent manner, whereas the M_2_ antagonist methoctramine blocks vagus nerve-evoked bradycardia (65). Collectively, the widely distributed M_2_-AChR exerts multifaceted modulatory effects across diverse cardiac physiological and pathological processes. Nevertheless, promising cardioprotective observations from pre-clinical animal models have not been fully translated into clinical practice. In the large-scale 2016 INOVATE-HF trial, therapeutic vagus nerve stimulation targeting parasympathetic modulation failed to reduce mortality or heart-failure-related clinical events in patients with heart failure, highlighting the translational gap between pre-clinical findings and human therapeutic outcomes (66).

## 4 Calcium release pathways

### 4.1 L-type calcium channels (LTCCs)

LTCCs are voltage-gated ion channels modulated by shifts in transmembrane potential. Characterized by slow activation kinetics and prolonged open time, these channels constitute the major pathway for extracellular Ca^2+^ entry during excitation and are tightly coupled to cardiomyocyte E-C coupling. LTCCs assemble as large macromolecular complexes composed of *α*_1_, *α*_2_, β, *γ* and *δ* subunits. The cardiac *α*_1_C subunit (Cav1.2), expressed as 240 kDa and 210 kDa isoforms in adult ventricular myocytes, contains four homologous transmembrane domains and localizes to the plasma membrane (67). Cav1.2 shapes action potential morphology, initiates E-C coupling, and governs the velocity and magnitude of myocardial contraction, serving as an indispensable regulator of cytosolic calcium homeostasis. Upon membrane depolarization, minimal Ca^2+^ permeates through Cav1.2 to bind and activate RyR2, triggering robust Ca^2+^ efflux from SR stores via calcium-induced calcium release (CICR) (67). Following Ca^2+^ entry, Cav1.2 undergoes two distinct inactivation modes: voltage-dependent inactivation dominates under basal conditions via negative feedback triggered by Ca^2+^ influx and SR calcium release, whereas Ca^2+^-dependent inactivation prevails after β-adrenergic receptor stimulation (68). β_1_-AR signaling potentiates LTCC-mediated Ca^2+^ influx to elicit positive inotropic responses (69). Consequently, alterations in LTCC expression density and function profoundly remodel cardiac contractile performance.

Pathogenic mutations in LTCC-encoding genes augment channel open probability, triggering cardiomyocyte hyperexcitability, arrhythmia and HF (70). In failing myocardium, defective LTCC phosphorylation and subcellular microdomain remodeling drive the translocation of functional channels from transverse tubules to surface sarcolemma (71). Multiple preclinical models, including tachycardia-induced canine HF, exhibit markedly diminished LTCC density-a central driver of systolic dysfunction (72–74). Single-channel patch-clamp recordings reveal that LTCCs display higher open probability in failing myocardium relative to healthy tissue. This adaptive remodeling compensates for reduced total channel abundance at low heart rates to sustain physiological calcium transients and contraction; however, such compensation collapses under rapid cardiac pacing (75, 76). Overexpression of the LTCC β_2_a subunit elevates channel open probability and facilitates membrane trafficking of *α*_1_C subunits to amplify Ca^2+^ influx (77). Consistent with this microdomain shift, excessive CaMKII-dependent phosphorylation of crest-residing LTCCs elevates channel open probability in failing hearts, triggering disrupted intracellular calcium cycling and arrhythmogenic calcium oscillations (71). Beyond modifying channel open probability via mode 2 gating of Cav1.2, CaMKII-dependent phosphorylation on the *α*_1_C and β_2_a subunits also augments sustained late Ca^2+^ window current during the action potential plateau. The prolonged inward Ca^2+^ flux lengthens action potential duration and creates a proarrhythmic electrophysiological substrate that readily generates early afterdepolarizations (EADs), which serve as major triggers of ventricular arrhythmia in failing cardiomyocytes (78). It is worth noting that while LTCC protein density is reduced in human heart failure with reduced ejection fraction (HFrEF) despite maintained basal L-type calcium current (73), studies focusing on T-tubule architecture and calcium transients indicate relatively preserved LTCC-associated calcium signaling in HF with preserved ejection fraction (HFpEF) (79). Of note, profound LTCC-RyR2 microdomain uncoupling is a hallmark of HFrEF, while such dyadic disruption is not prominent in HFpEF; direct head-to-head human myocardial comparisons across both phenotypes remain limited (80).

LTCC blockers are widely applied in clinical cardiovascular therapeutics. Such agents limit extracellular Ca^2+^ entry, suppress early afterdepolarizations, and reduce ventricular arrhythmia incidence. However, they simultaneously disrupt normal E-C coupling and exert negative inotropic effects. Notably, two non-dihydropyridine agents, verapamil and diltiazem, are conventional LTCC blockers with potent negative-inotropic properties; therefore, they are generally contraindicated in patients with HFrEF owing to the risk of further depressing myocardial contractility (81). The purine derivative roscovitine selectively inhibits late Ca^2+^ current without impairing myocardial contraction by reshaping Cav1.2 gating kinetics (82). Cav1.2 remains a high-priority intervention target for multiple cardiac disorders and warrants further in-depth translational investigations (83).

### 4.2 RyR2 and calstabin regulatory proteins

Ryanodine receptors (RyRs) constitute the largest known ion channel superfamily, with a molecular mass of approximately 565-kDa (84). Three mammalian isoforms exist: RyR1 (predominantly expressed in skeletal muscle and nervous tissue), RyR3 (enriched in the brain and specialized muscle), and RyR2 (selectively abundant in cardiac myocytes alongside brain tissue) (84). Cardiac RyR2 subunits oligomerize into homotetrameric complexes anchored to sarcoplasmic reticulum (SR) membranes, whose coordinated gating governs systolic Ca^2+^ release and sustains basal cardiomyocyte contractile function (85). Multiple kinases, phosphatases, and calmodulin collectively tune RyR2 Ca^2+^ sensitivity; post-translational modifications of these regulatory partners trigger domain conformational rearrangements to switch the channel between open and closed states (86). Ca^2+^ influx induced by membrane depolarization binds cytosolic RyR2 and activates channel opening, initiating massive SR calcium release during E-C coupling.

Calstabin1 (FKBP12) and Calstabin2 (FKBP12.6) stabilize RyR macromolecular assemblies and suppress spontaneous diastolic calcium leakage. FKBP12 tends to preferentially associate with skeletal RyR1, while FKBP12.6 generally exhibits higher binding affinity for cardiac RyR2 (87, 88). Despite lower myocardial abundance relative to FKBP12, FKBP12.6 possesses higher binding affinity toward RyR2, tending to lock the channel in a closed conformation during diastole and limit unregulated SR Ca^2+^ efflux (89). Congenital mutations or acquired functional defects disrupting RyR2–FKBP12.6 interactions induce asynchronous myocardial contraction and accelerate HF progression (90, 91). Under HF conditions, persistent sympathetic hyperactivity elevates circulating catecholamine levels and activates protein kinase A (PKA). Excessive PKA-mediated RyR2 phosphorylation dissociates the RyR2–FKBP12.6 complex and impairs channel gating function (24). Beyond PKA-dependent phosphorylation, Ca^2+^/calmodulin-dependent kinase II (CaMKII) represents another vital signaling pathway regulating RyR2 under adrenergic stimulation. Sympathetic stimulation raises cytosolic calcium concentrations, which drives the formation of Ca^2+^-bound calmodulin (CaM). This complex further triggers autophosphorylation of CaMKII to sustain its catalytic activity. Once activated, CaMKII phosphorylates RyR2 at Ser2814, a residue distinct from the canonical PKA phosphorylation site Ser2808. This specific post-translational modification promotes severe pathological diastolic sarcoplasmic reticulum calcium leak, and this regulatory cascade functions independently of PKA signaling (20, 21, 92). Notably, the calcium-leak effect driven by Ser2814 hyperphosphorylation is prominent in nonischemic heart failure, while this alteration is less pronounced in ischemic cardiac injury (21). Clinical myocardial specimens from patients with terminal heart failure further confirm that sustained overactivation of CaMKII triggers persistent sarcoplasmic reticulum calcium cycling defects in diseased cardiomyocytes (93). As an intrinsic allosteric modulator stably bound to RyR2, CaM exerts prominent inhibitory effects on resting channel gating, with its regulatory function dependent on the phosphorylation status of RyR2. Under physiological resting conditions, bound CaM restrains excessive RyR2 opening. However, adrenergic overstimulation leads to heavy RyR2 phosphorylation, weakening the binding affinity between RyR2 and CaM and eliminating this intrinsic inhibitory effect. Existing review work has summarized that impaired CaM-RyR2 binding is a shared pathological feature across multiple heart failure subtypes (94). Single-channel recordings on human RyR2 channels (both native failing cardiac RyR2 and recombinant mutant variants) further clarified this phosphorylation-dependent regulatory pattern. The study demonstrated that CaM can only suppress diastolic calcium release when either the Ser2808 or Ser2814 residue of RyR2 is phosphorylated, establishing a unique CaM regulator*y* axis activated under adrenergic stress in failing human myocardium (95).

These phosphorylation-dependent modifications together disrupt intramolecular contacts within RyR2. Disrupted intradomain interactions within RyR2 further exacerbate diastolic SR calcium leakage (96). Uncontrolled persistent RyR2 opening triggers sustained calcium efflux, depletes SR calcium stores, blunts myocardial contractility and generates arrhythmogenic cytosolic calcium oscillations (97). Accumulated experimental data demonstrate that reduced cellular FKBP12.6 abundance and its pathological dissociation from RyR2 alter channel conformation, attenuating contractile performance and elevating risks of ventricular arrhythmia and sudden cardiac death (98). Notably, the degree of RyR2 hyperphosphorylation and leak varies between HF phenotypes; it is typically robust in HFrEF but tends to be milder in HFpEF, suggesting that the therapeutic window for RyR2 stabilizers may differ based on underlying calcium cycling signatures.

RyR2 stands out as a high-priority intervention candidate for HF. Novel RyR2 stabilizers can eliminate pathological SR calcium leakage. However, selective RyR2 inhibitors remain unavailable for routine clinical application. JTV519 (K201), a benzothiazine derivative, represents the first compound validated to normalize aberrant RyR2 gating kinetics. In canine HF models, JTV519 reinforces the binding affinity between FKBP12.6 and RyR2, restrains spontaneous diastolic SR calcium leakage, preserves systolic and diastolic contractile performance and mitigates pathological ventricular remodeling (99). The cardioprotective properties of JTV519 are abrogated in FKBP12.6 knockout mice, verifying that its protective effects depend on strengthened calstabin–RyR2 interactions (100, 101).

In addition to JTV519, several conventional clinical drugs have been shown to modulate RyR2 function and alleviate calcium cycling disorders in failing hearts. As a classic agent used for malignant hyperthermia, dantrolene exerts direct protective effects on diseased cardiomyocytes by stabilizing interdomain interactions of RyR2, which corrects abnormal channel opening and improves impaired contractile function (102). Accumulated mechanistic evidence further demonstrates that dantrolene suppresses diastolic SR calcium leak and arrhythmogenic calcium events under pathological conditions with excessive CaMKII activity, thereby mitigating CaMKII-driven atrial arrhythmia susceptibility (103). Additional translational research elaborates that dantrolene exerts selective inhibitory effects only on dysfunctional RyR2 rather than normal cardiac channels, and its core protective mechanism involves restoring physiological calmodulin binding to defective RyR2. Nevertheless, chronic oral dantrolene carries potential hepatotoxicity, which greatly limits its long-term administration for chronic heart failure management (104). Flecainide, a widely used class-I antiarrhythmic drug, modulates RyR2 gating in a concentration-dependent manner independent of its sodium channel-blocking effects. Recent functional studies have clarified its complex regulatory roles in restraining excessive RyR2-mediated calcium release, which accounts for its efficacy against adrenergic-induced ventricular arrhythmias under adrenergic-stressed conditions (105, 106). Phenytoin also exhibits direct inhibitory effects on overactive RyR2 channels. Single-channel recordings confirm that phenytoin reduces RyR2 open probability and attenuates exaggerated SR calcium efflux specifically in failing human cardiomyocytes, with no obvious suppression of RyR2 derived from healthy myocardium. Such selective inhibitory profile endows phenytoin with favorable cardioprotective potential and better biosafety compared with hepatotoxic dantrolene. Nevertheless, clinical evidence validating its routine use in chronic heart failure therapy remains insufficient (107). Collectively, these RyR2-modulating drugs yield consistent benefits in preclinical models, whereas their clinical translation for heart failure treatment still requires further investigation.

Reversing excessive RyR2 phosphorylation constitutes a core therapeutic mechanism underlying β-blocker treatment for HF (108). Clinical myocardial tissue analyses reveal that β-blocker therapy restores homeostatic balance of the RyR2 macromolecular complex, attenuates RyR2 hyperphosphorylation, and reinforces FKBP12.6 binding in failing human myocardium to improve contractile function (92, 109). Specifically, propranolol suppresses excessive RyR2 phosphorylation, blocks FKBP12.6 dissociation, eliminates calcium leakage, and attenuates progressive ventricular remodeling (110). Gene therapy targeting FKBP12.6 upregulation provides an alternative strategy: adenovirus-mediated FKBP12.6 overexpression stabilizes the RyR2 closed conformation, synchronizes SR calcium release events, amplifies calcium transients and strengthens myocardial contraction (111). Ultrasound microbubble-mediated FKBP12.6 gene transfection alleviates cardiomyocyte injury and improves left ventricular ejection fraction and fractional shortening in canine HF models (112). Collectively, these preclinical mechanistic findings lay a solid foundation for developing targeted calcium-regulating therapies against HF.

## 5 Calcium reuptake machinery

SERCA2a and phospholamban (PLB, its endogenous inhibitory peptide) constitute the core molecular complex responsible for diastolic sarcoplasmic reticulum calcium recycling, representing the primary pathway to clear cytosolic Ca^2+^ post-systole and maintain normal diastolic relaxation (113). Sarco/endoplasmic reticulum Ca^2+^-ATPase (SERCA) constitutes a family of monomeric transmembrane ATPases that translocate cytosolic Ca^2+^ back into the SR against concentration gradients. Multiple splice variants exist within the SERCA family; SERCA2a represents the cardiac-specific isoform, whose catalytic activity exceeds that of non-cardiac subtypes by 10-to-50 fold (114). SERCA2a-mediated SR calcium sequestration determines the rate of myocardial relaxation and acts as a central modulator of cytosolic calcium homeostasis, proliferation, and survival. PLB is a small SR membrane polypeptide consisting of 52 amino acid residues, which assembles into pentameric complexes under resting conditions (115). As the primary endogenous suppressor of SERCA2a activity, PLB binds the pump reversibly and reduces its intrinsic Ca^2+^ binding affinity (116, 117).

SERCA2a possesses two functionally critical cytoplasmic domains: a phosphorylation-domain segment (residues 336–412) involved in allosteric communication underlying PLB-dependent regulation, and a nucleotide-binding domain (residues 467–762) dictating the pump's Ca^2+^ affinity (117, 118). Phosphorylation of PLB triggers its dissociation from SERCA2a, abolishes inhibitory constraints, and accelerates SR calcium retrieval to facilitate myocardial relaxation. Elevated cytosolic Ca^2+^ further boosts SERCA2a's Ca^2+^ affinity via positive feedback loops (119, 120). In failing cardiomyocytes, SR calcium dyshomeostasis serves as a core pathological hallmark, primarily manifested as depleted SR calcium pools (121). Reduced SERCA2a protein abundance and catalytic activity jointly disrupt SR calcium retention—a conserved molecular signature observed across various HF phenotypes (122–124). Hypophosphorylation of PLB strengthens its inhibitory effect on SERCA2a. When SERCA2a expression is downregulated against constant PLB levels, the elevated PLB/SERCA2 ratio lowers the calcium affinity of the SERCA pump and worsens systolic dysfunction—a consistent phenotype verified in multiple HF animal models (125, 126). However, the nature of SERCA2a-related inhibition differs among HF subtypes. In HFrEF, reduced SERCA2a protein expression is a predominant molecular feature. By contrast, in HFpEF, SERCA2a expression is often maintained, though its activity can be markedly suppressed due to PLB hypophosphorylation, despite inter-study variability in human myocardial observations (127). Therefore, boosting SERCA2a activity has become a pivotal therapeutic strategy for HF management.

Traditional catecholamine-based positive inotropes increase PLB phosphorylation yet are associated with well-documented long-term clinical limitations, prompting the development of selective SERCA2a-targeted therapeutics to mitigate such drawbacks (10). Istaroxime, an intravenous drug indicated for short-term treatment of decompensated HF, relieves PLB-dependent suppression of SERCA2a, accelerates SR calcium reuptake and enhances global myocardial contractility (128, 129). SERCA2a activity can also be modulated by diverse post-translational modifications. For instance, ginsenoside Rg3 augments SERCA2a SUMOylation to restore impaired cardiomyocyte function, representing a low-toxicity natural therapeutic candidate (130). Adenovirus-mediated SERCA2a overexpression expands SR calcium reserves, ameliorates bidirectional cardiac function, curbs cardiomyocyte death, and inhibits arrhythmogenesis (131). SERCA2a gene therapy stabilizes SR calcium loading, reduces RyR2 hyperphosphorylation, blunts diastolic calcium leak and ventricular arrhythmias, and mitigates myocardial injury and progressive cardiac remodeling (132, 133). Nevertheless, phase II trials have yielded promising preliminary outcomes yet demonstrated inconsistent efficacy across large patient cohorts, and several unresolved challenges hinder the clinical translation of SERCA2a gene therapy regarding long-term safety and sustained therapeutic benefits (134–136).

## 6 Other key calcium regulators

### 6.1 Cardiac troponin C (cTnC)

Cardiac troponin C (cTnC), cardiac troponin I (cTnI) and cardiac troponin T (cTnT) assemble into the intact cardiac troponin complex, which transduces cytosolic Ca^2+^ signals into mechanical contraction of myofilaments (137). As the primary Ca^2+^-sensing subunit within this complex, cTnC dictates the magnitude of myocardial contractile force (138). cTnI suppresses actin-myosin ATPase activity to maintain diastolic relaxation (139), whereas cTnT anchors the entire complex to tropomyosin filaments (140). cTnC contains four conserved EF-hand structural motifs forming two globular domains linked by a flexible regulatory segment: an N-terminal low-affinity domain that interacts with cTnI, and a C-terminal high-affinity Ca^2+^-binding domain (137). Ca^2+^ released from the SR binds cTnC, inducing conformational rearrangement that displaces tropomyosin and unmasks actin binding sites, enabling cross-bridge cycling and myocardial contraction. As cytosolic Ca^2+^ concentration declines during diastole, Ca^2+^ dissociates from cTnC, allowing myocardial relaxation (137, 141).

Pathogenic mutations in troponin-encoding genes trigger various cardiomyopathies. Structural remodeling of cTnC weakens its intrinsic Ca^2+^ binding capacity, blunting myofilament Ca^2+^ sensitivity and reducing contractile performance in failing hearts (142). Altered cTnC structure and Ca^2+^ affinity directly impair myofilament Ca^2+^ responsiveness and exacerbate myocardial contractile dysfunction during heart failure progression. Small-molecule agents targeting troponin-mediated Ca^2+^ sensitivity constitute high-priority intervention candidates for HF (138). Levosimendan, a calcium sensitizer approved for managing acute decompensated systolic HF in Europe, binds the hydrophobic pocket within the N-terminus of cTnC. This interaction enhances myofilament responsiveness to Ca^2+^ without elevating global cytosolic Ca^2+^ levels, thereby boosting cardiac output and ameliorating HF clinical manifestations (143). However, the utility of such agents must be carefully evaluated against the underlying phenotype; while they may improve acute hemodynamics in systolic failure, elevated resting cytosolic Ca^2+^ in certain HFpEF contexts may limit their long-term efficacy. Subsequent translational research should prioritize novel troponin-targeted compounds and combinatorial multi-target therapeutic regimens.

### 6.2 Na^+^/Ca^2+^ exchanger 1 (NCX1)

The NCX family comprises bidirectional plasma membrane transporters that coordinate cytosolic Na^+^ and Ca^2+^ homeostasis, with three mammalian isoforms characterized to date: NCX1 (widely expressed in the heart, brain and kidney), neuron-restricted NCX2, and NCX3 (abundant in the brain and skeletal muscle) (144). Cardiac NCX1 mediates the exchange of one cytosolic Ca^2+^ for three extracellular Na^+^, participating in cardiomyocyte repolarization and E-C coupling (145, 146). Under physiological resting conditions, NCX1 predominantly operates in the forward mode to extrude cytosolic Ca^2+^ during diastole, whereas reverse-mode Ca^2+^ influx only under specific pathological conditions. End-stage HF triggers transcriptional and translational remodeling that upregulates NCX1 mRNA and membrane protein abundance, serving as a compensatory response to impaired SERCA2a-mediated calcium reuptake (147). Although augmented forward NCX1 activity partially mitigates cytosolic calcium overload, sustained excessive Ca^2+^ extrusion depletes SR calcium stores and aggravates systolic contractile dysfunction. Persistent activation of reverse-mode NCX1 transport drives massive Ca^2+^ entry into cardiomyocytes, provoking severe cytosolic calcium overload, myocyte injury, and ventricular arrhythmias (148–150). Accordingly, targeted suppression of NCX1 function via gene delivery or small-molecule pharmaceuticals emerges as a feasible translational strategy for HF and other cardiovascular disorders.

Consistent evidence from chronic HF animal models demonstrates that acute NCX1 inhibition shortens action potential duration, reduces spatial repolarization heterogeneity, and suppresses polymorphic ventricular tachycardia incidence (151, 152). The selective NCX inhibitor SEA0400 blocks pathological reverse-mode Ca^2+^ transport, attenuates ventricular interstitial fibrosis, and improves long-term survival across HF animal models (153). Cardiac contractility-modulating signals normalize aberrant NCX1 expression and phosphorylation levels in failing canine myocardium, restoring balanced cytosolic calcium cycling and improving left ventricular contractile performance (154). Collectively, these preclinical data validate NCX1 as a high-priority intervention candidate for HF treatment.

### 6.3 Mitochondrial permeability transition pore (MPTP)

Mitochondria represent the primary organelles responsible for energy metabolism in cardiomyocytes, and they also serve as critical hubs for intracellular calcium homeostasis (155). Prior to accessing inner mitochondrial membrane transporters, cytosolic Ca^2+^ must cross the outer mitochondrial membrane through voltage-dependent anion channels (VDAC). Rather than simply forming non-selective passive pores, VDAC can dynamically adjust mitochondrial permeability to Ca^2+^ (156). Two key calcium-transporting machineries reside within the inner mitochondrial membrane: the mitochondrial calcium uniporter (mtCU) complex and the mitochondrial Na^+^-Ca^2+^-Li^+^ exchanger (NCLX). The mtCU complex mediates Ca^2+^ influx from cytosol into the mitochondrial matrix, assembled by the pore-forming subunit MCU, essential auxiliary subunit EMRE, and regulatory gating subunits MICU1 and MICU2 (157). Importantly, mtCU function varies dramatically across tissues. Cardiomyocytes contain far fewer open mtCU channels and display lower overall mtCU conductance compared with skeletal muscle cells. Although cardiac MICU1 expression is relatively low, MICU1-and MICU2-dependent gating is still operational in human and murine cardiomyocytes, and cardiac mtCU only shows attenuated responses to global cytosolic calcium elevations, creating a tissue-unique pattern of mitochondrial calcium handling (157, 158). Encoded by the SLC8B1 gene, NCLX serves as the dominant transporter responsible for Ca^2+^ efflux from cardiac mitochondrial matrix. It mediates electrogenic ion exchange, exporting one matrix Ca^2+^ in exchange for three cytosolic Na^+^; lithium can substitute for sodium to sustain transport activity, a structural feature that distinguishes mitochondrial NCLX from the plasma membrane NCX isoform (159). Under physiological conditions, NCLX continuously counterbalances mtCU-driven Ca^2+^ influx to restrict excessive matrix Ca^2+^ accumulation, maintains stable mitochondrial energy metabolism, and limits basal reactive oxygen species generation. A cardiac-specific feature of NCLX is its spatial colocalization with sarcoplasmic reticulum SERCA and RyR2 channels. This microdomain arrangement enables bidirectional calcium crosstalk between mitochondria and sarcoplasmic reticulum, coordinating global cardiomyocyte calcium cycling and cardiac electrical rhythmicity (159, 160).

Following Ca^2+^ permeation through outer-membrane VDAC, coordinated activity of mtCU and NCLX balances mitochondrial Ca^2+^ influx and efflux, aligns tricarboxylic acid (TCA) cycle-derived ATP synthesis with fluctuating cardiac workload, and modulates cytosolic Ca^2+^ transients. Matrix Ca^2+^ also links mitochondrial calcium cycling to cellular redox homeostasis by modulating the synthesis and clearance of ROS (161). Beyond short-term regulation of energy production, long-term shifts in mtCU and NCLX activity indirectly alter electron transport chain efficiency, determine cardiac substrate utilization profiles, and shape the capacity of cardiomyocytes to withstand physiological stress. Sustained impairment of these two transporters disrupts oxidative phosphorylation and accelerates pathological ventricular remodeling in heart failure (162). In failing myocardium, chronically elevated intracellular Na^+^ strongly inhibits NCLX-mediated Ca^2+^ extrusion, and this inhibitory effect emerges well before measurable MPTP opening (159). Impaired NCLX transport disrupts the mtCU-NCLX calcium equilibrium, leading to progressive mitochondrial matrix calcium overload. Excess matrix Ca^2+^ amplifies oxidative stress, further reduces the threshold for MPTP opening, and eventually triggers sustained pathological MPTP activation to initiate cardiomyocyte damage and death (162).

The MPTP functions as a master molecular switch governing mitochondrial homeostasis and cardiomyocyte survival (155). The MPTP is a non-selective inner mitochondrial membrane channel permeable to molecules smaller than 1.5-kDa. Early models proposed the complex of voltage-dependent anion channel (VDAC), adenine nucleotide translocase (ANT) and cyclophilin D (CyP-D) as the core pore-forming machinery; nevertheless, the precise molecular composition of the MPTP remains highly controversial. Accumulated evidence confirms that CyP-D functions only as an essential regulatory cofactor rather than an integral pore subunit, and definitive proof supporting VDAC and ANT as indispensable structural components of the MPTP is still lacking (163, 164). Recent biochemical, cryo-electron microscopy and genetic data suggest the F_1_F₀ ATP synthase complex is a major candidate for MPTP pore assembly, and its c-subunit ring is thought to form the core inner membrane channel (165). However, contradictory experimental evidence persists for this structural model (166).

Dysregulated mitochondrial homeostasis accelerates HF progression (167), with MPTP opening serving as a central mediator linking myocardial dysfunction to irreversible cardiomyocyte death. Transient MPTP activation constitutes a physiological regulatory cascade: it facilitates Ca^2+^ efflux from the mitochondrial matrix to stabilize the intramitochondrial microenvironment and coordinate global cellular metabolic activity (168). Pathological Ca^2+^ accumulation within the mitochondrial matrix—often accompanied by excessive oxidative stress and elevated inorganic phosphate—triggers aberrant sustained MPTP activation. Persistent MPTP opening represents a pathological hallmark that initiates a cascade of detrimental cellular events (169). Long-term activation suppresses mitochondrial ATP synthesis and depletes cellular energy stores, disrupting basal metabolic balance. Increased permeability of the inner mitochondrial membrane allows uncontrolled leakage of small molecules (including Ca^2+^), disturbing cytosolic ion homeostasis and triggering mitochondrial swelling alongside subsequent rupture of the outer mitochondrial membrane. This sequential pathological cascade ultimately drives cardiomyocyte apoptosis or necrotic death (163).

The MPTP has been identified as a core therapeutic target for myocardial protection against ischemic and failing heart conditions (170). Modulating MPTP activity exerts cardioprotective effects via two distinct pathways: indirect relief of cytosolic calcium overload and oxidative stress, or direct suppression of pore opening through targeted small-molecule antagonists (171). Cumulative preclinical data and limited clinical observations demonstrate that MPTP inhibitors reduce infarct size, mitigate cardiomyocyte loss, and attenuate progressive ventricular remodeling in HF settings (172). Nevertheless, the translational efficacy of cyclosporine A, the classic cyclophilin D/MPTP inhibitor, was challenged by the large-scale CIRCUS multicenter trial, which enrolled 970 patients with anterior ST-elevation myocardial infarction. Intravenous cyclosporine administered prior to percutaneous coronary intervention failed to lower the composite endpoint of all-cause mortality, heart failure rehospitalization, and adverse left ventricular remodeling at 1-year follow-up, despite robust preclinical cardioprotective evidence (173). As an alternative candidate, NIM811 is a non-immunosuppressive cyclosporine analog that retains potent MPTP-blocking activity without calcineurin inhibition. It exerts protective effects against MPTP-mediated mitochondrial injury across distinct non-cardiac preclinical models, including collagen VI congenital muscular dystrophy (174) and hepatic ischemia-reperfusion injury (175), and 14-day phase II human clinical trials in HCV-infected patients have confirmed its favorable overall safety tolerance across multiple oral doses (176). Although dedicated clinical investigations of NIM811 for heart disease remain absent, this compound provides a promising novel chemical scaffold to circumvent the clinical limitations of cyclosporine. Collectively, while the first-generation MPTP inhibitor cyclosporine showed disappointing clinical outcomes, novel agents such as NIM811 preserve the core mitochondrial protective benefits of MPTP suppression. Therefore, therapeutic strategies targeting MPTP still hold substantial untapped potential to halt progressive cardiac decompensation in heart failure.

## 7 Summary

**Figure 2:**
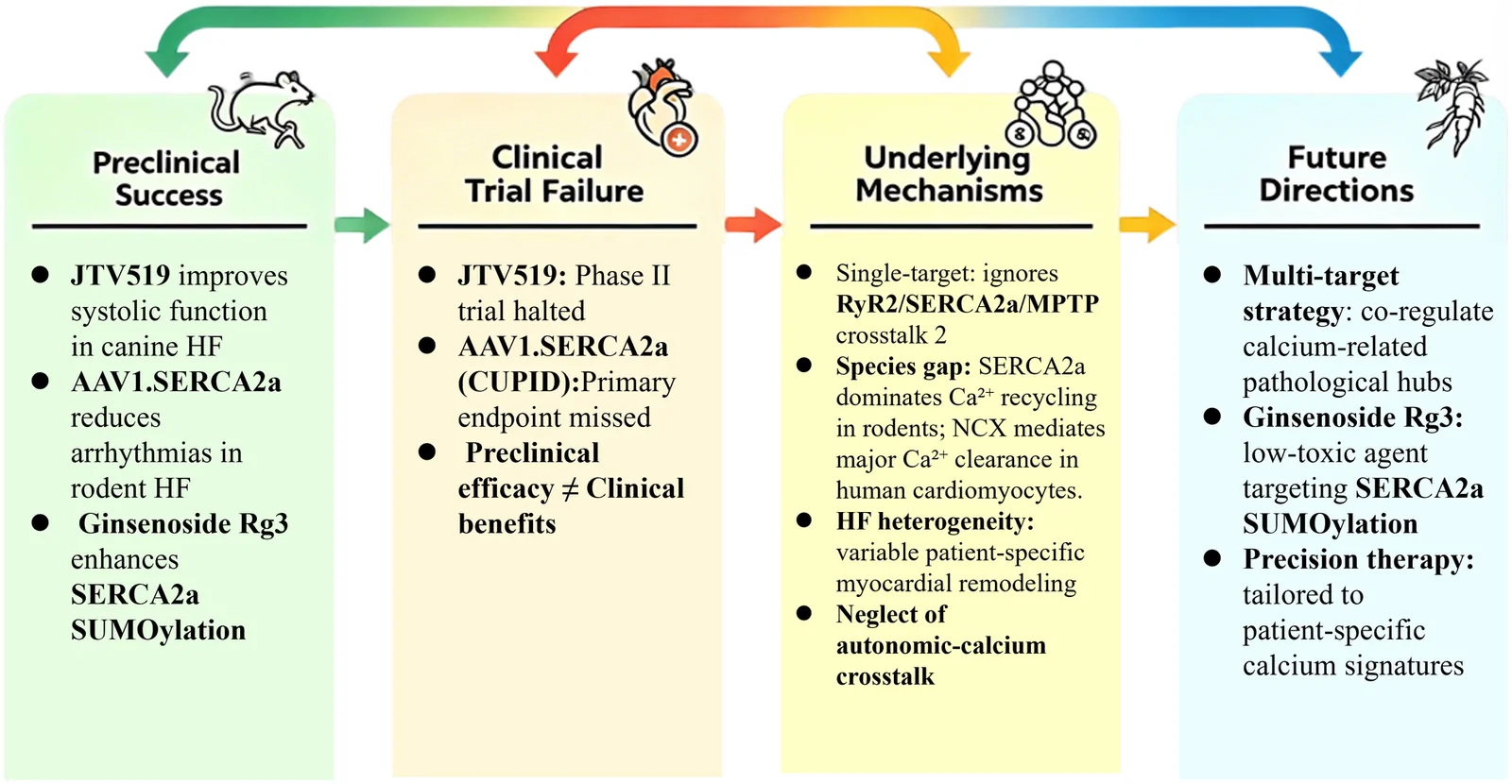
Translational roadmap of calcium-targeted therapies for heart failure. Preclinical efficacy of RyR2 stabilizers (JTV519) and SERCA2a gene therapy (AAV1.SERCA2a) fails to translate to clinical benefit, due to four core translational barriers: single-target limitations, prominent species gap in cardiomyocyte calcium handling (SERCA2a dominates Ca^2+^ recycling in rodents, while NCX undertakes major Ca^2+^ clearance in human myocardium), heterogeneous myocardial remodeling across HF patients, and neglected autonomic-calcium crosstalk. Future directions prioritize multi-target approaches, low-toxicity natural compounds such as ginsenoside Rg3 (promotes SERCA2a SUMOylation, denoted by the ginseng icon), and precision medicine tailored to individual calcium remodeling profiles.

HF represents the end-stage phenotype of heterogeneous cardiovascular diseases and remains an unmet clinical challenge globally. Current therapeutic regimens primarily alleviate clinical symptoms yet cannot arrest progressive myocardial damage. Ca^2+^ acts as an indispensable intracellular second messenger coupling cardiomyocyte electrical excitation to mechanical contraction. Calcium handling coordinated by β_1_-AR and M_2_-AChR signaling axes forms the fundamental molecular network controlling systolic and diastolic cardiac performance. HF disturbs nearly all segments of calcium signaling cascades, collectively inducing systolic dysfunction and life-threatening ventricular arrhythmias. The translational roadmap of calcium-targeted therapies, highlighting key clinical bottlenecks and future precision strategies, is illustrated in Figure 2.

Numerous translational obstacles limit the clinical transformation of calcium-modulating therapies. The RyR2 stabilizer JTV519 delivers robust cardioprotection across diverse preclinical HF models. However, consistent therapeutic benefits have not been uniformly reproduced in human trials. Similarly, SERCA2a gene therapy generates conflicting safety and efficacy data across different patient subgroups. A prominent divide exists, hindering the broad clinical application of single-molecule calcium regulators. While distinct calcium remodeling signatures exist across the HF spectrum (e.g., between HFrEF and HFpEF), therapeutic strategies must ultimately aim to correct specific molecular lesions present in individual patients rather than adopting a uniform approach.

Restoring physiological calcium balance offers a feasible therapeutic route to reverse pathological ventricular remodeling and optimize long-term prognosis in HF patients. Future translational investigations should prioritize multi-target combination schemes that concurrently regulate RyR2, SERCA2a and MPTP to re-establish whole-cell calcium homeostasis. Notably, natural bioactive components such as ginsenoside Rg3 alleviate calcium dysregulation by boosting SERCA2a SUMOylation, providing low-toxic candidate adjuvant agents requiring in-depth mechanistic and clinical verification. Multi-axis calcium regulatory interventions attenuate cardiomyocyte injury and reverse ventricular remodeling, yielding durable clinical benefits for HF cohorts.

## 8 Limitations of current research

Multiple gaps remain in current research investigating dysregulated myocardial calcium cycling in HF. First, the bulk of mechanistic evidence is derived from rodent, canine, and zebrafish preclinical models. These species exhibit divergent calcium-handling kinetics, distinct autonomic signaling regulation, and dissimilar disease progression trajectories, failing to faithfully recapitulate human HF pathophysiology and producing persistent translational disconnects between bench findings and clinical trial observations (177). Emerging human-derived translational models, including human induced pluripotent stem cell-derived cardiomyocytes (hiPSC-CMs) and cardiac organoids, provide complementary experimental platforms to narrow this translational gap between animal-based mechanistic studies and clinical trials. Nevertheless, these human-derived experimental systems still bear inherent drawbacks: stem-cell-based models suffer from incomplete cellular maturation, while all of these platforms face constraints related to tissue complexity and practical experimental accessibility (178, 179). Second, most available research adopts single-target experimental frameworks, whereas crosstalk between LTCC, RyR2, SERCA2a, NCX1 and MPTP under pathological stress remains insufficiently defined; head-to-head comparisons of multi-target combinatorial therapies across HF populations are rarely reported. Given the interconnected nature of cardiomyocyte calcium homeostasis, combinatorial multi-target regimens represent a promising therapeutic direction. For instance, RyR2-modulating agents such as dantrolene, flecainide, and phenytoin could be combined with therapeutic strategies that modulate SERCA2a or NCX1 function to cooperatively restore intracellular calcium homeostasis in failing cardiomyocytes; nevertheless, optimal drug combinations, safe dosage windows, and potential off-target risks for such combinatorial strategies remain largely underexplored (180). Third, clinical trials testing calcium-modulating agents are predominantly short-term phase II investigations with small sample cohorts. Long-term follow-up data tracking cardiovascular mortality and arrhythmic adverse events remain scarce, particularly for novel SERCA2a gene therapy and natural bioactive compounds such as ginsenoside Rg3. Fourth, ethnic and geographic disparities in cardiac calcium remodeling signatures are poorly elucidated: most human myocardial tissue datasets are collected from European and North American cohorts, while large multi-omics profiling datasets characterizing calcium homeostasis alterations in Chinese HF patients are lacking. Last, few studies systematically characterize progressive shifts in calcium handling defects throughout distinct HF stages, and stage-tailored therapeutic regimens have not yet been established. Future translational work should combine multi-omics profiling of human cardiac specimens, large longitudinal clinical cohorts, and multi-target combinatorial preclinical models to address these gaps and facilitate the development of precision calcium-modulating therapeutics for HF.
